# Cross-cultural adaptation and psychometric properties of the Chinese version of the Person-Centered Maternity Care Scale

**DOI:** 10.1186/s12884-023-05959-x

**Published:** 2023-09-09

**Authors:** Xiaoying Zhong, Rong Hu, Patience A. Afulani, Xixi Li, Xiujing Guo, Tingting He, Dehua Li, Zuowei Li

**Affiliations:** 1 Department of Nursing, School of Medicine, Mianyang Central Hospital, University of Electronic Science and Technology of China, Mianyang, China; 2 Department of Nursing, School of Medicine, Mianyang Central Hospital, University of Electronic Science and Technology of China, Mianyang, China; 3 Department of Epidemiology and Biostatistics, University of California, San San Francisco, California USA; 4 Department of Nursing, School of Medicine, Mianyang Central Hospital, University of Electronic Science and Technology of China, Mianyang, China Philippines Women’s University, Manila, Philippines; 5 Department of Nursing, West China Second University Hospital, Sichuan University / Laboratory of Birth Defects and Related Diseases of Women and Children (Sichuan University), Ministry of Education, Chengdu, Sichuan China; 6 Nephrology department, The Third Hospital of Mianyang, Sichuan Mental Health Center/ The Third Hospital of Mianyang (Sichuan Mental Health Center), Mianyang, China; 7 Department of Nursing, West China Second University Hospital, Sichuan University / Laboratory of Birth Defects and Related Diseases of Women and Children (Sichuan University), Ministry of Education, Chengdu, Sichuan China; 8 Department of Nursing, The Third Hospital of Mianyang, Sichuan Mental Health Center/ The Third Hospital of Mianyang (Sichuan Mental Health Center), Mianyang, China

**Keywords:** Person-centered maternity care, Quality of care, Psychometric properties, Maternity care, Chinese adaptation

## Abstract

**Background:**

Increasing evidence show that women across the world face unacceptable mistreatment during childbirth. Person-centered maternity care is fundamental and essential to quality of healthcare services. The aim of this study was to translate and determine the psychometric properties of the Person-Centered Maternity Care (PCMC) Scale among Chinese postpartum women.

**Methods:**

A cross-sectional study was conducted among 1235 post-partum women in China. The cross-cultural adaptation process followed the Beaton intercultural debugging guidelines. A total of 1235 women were included to establish the psychometric properties of the PCMC. A demographic characteristics form and the PCMC were used for data collection. The psychometric properties of the PCMC were evaluated by examining item analysis, exploratory factor analysis, known-groups discriminant validity, and internal consistency.

**Results:**

The number of extracted common factors was limited to three (dignity & respect, communication & autonomy, supportive care), explaining a total variance of 40.8%. Regarding internal consistency, the Cronbach’s alpha coefficient and split-half reliability of the full PCMC score were 0.989 and 0.852, respectively.

**Conclusions:**

The Chinese version of the PCMC is a reliable and valid tool to assess person-centered care during childbirth in China.

## Background

Childbirth is one of the most significant and unique individual life events across the life course [1, 2], with around 9.56 million women in China giving birth in 2022 [3]. The World Health Organization (WHO) notes that person-centered maternity care is a fundamental and essential component of quality of care [4], and that all women should have access to high-quality maternity services [5–7]. Person-centered maternity care refers to care during childbirth that is respectful and responsive to individual women and their families’ preferences, needs, and values [8].

A significant body of evidence shows that women across the world face unacceptable mistreatment during childbirth [9]. A recent WHO-led study [10, 11] in four countries showed that more than one-third of women experienced mistreatment during childbirth in health facilities, including physical and verbal abuse, stigma and discrimination, failure to meet professional standards of care, and so on. Person-centered maternity care is important because having a negative experience is associated with disparities in adverse pregnancy and birth outcomes [12–14], as well as poorer mental health among postpartum women [15], such as postpartum depression, anxiety, and Post-traumatic Stress Disorder (PTSD) [16]. Furthermore, evidence suggests that improving person-centered maternity care enhances women’s trust in facility-based care while decreasing patient complaints and medical disputes [17, 18]. As such, it is imperative to have a good, valid, and reliable instrument for assessing levels of person-centered maternity care to inform effective strategies to improve maternal health services [6, 19, 20].

Several instruments have been developed to measure experience of care during the childbirth. Among these existing tools, are the revised Childbirth Experience Questionnaire (CEQ 2.0) [21], the Wijma Delivery Expectancy Questionnaire version A and B (WDEQ-A and WDEQ-B) [22, 23], which have been used by several researchers in China. The Person-Centered Maternity Care (PCMC) Scale is however the most comprehensive multidimensional measure of women’s person-centered experiences during childbirth compared to these measures [24, 25]. The Person-Centered Maternity Care (PCMC) Scale was developed based on a review of the literature and informed by the WHO Quality of Care Standards for improving Maternal and Newborn Health, resulting in a 30-item PCMC in Kenya [8].

To date, the PCMC has been translated and validated has been validated across multiple settings including low-, middle-, and high-income countries [4, 26], including in India [27], the United States [26], Turkey [24], and Cambodia [28], Sri Lanka [29], Nigeria [30], and Ethiopia [31, 32] where it has been found to have good psychometric properties. No study has, however, been conducted to assess the validity and reliability of PCMC in China.The purpose of this study is therefore to translate and determine the reliability and validity of the Chinese version of the PCMC with postpartum women in China.

## Methods

### Study design and participants

**Fig. 1:**
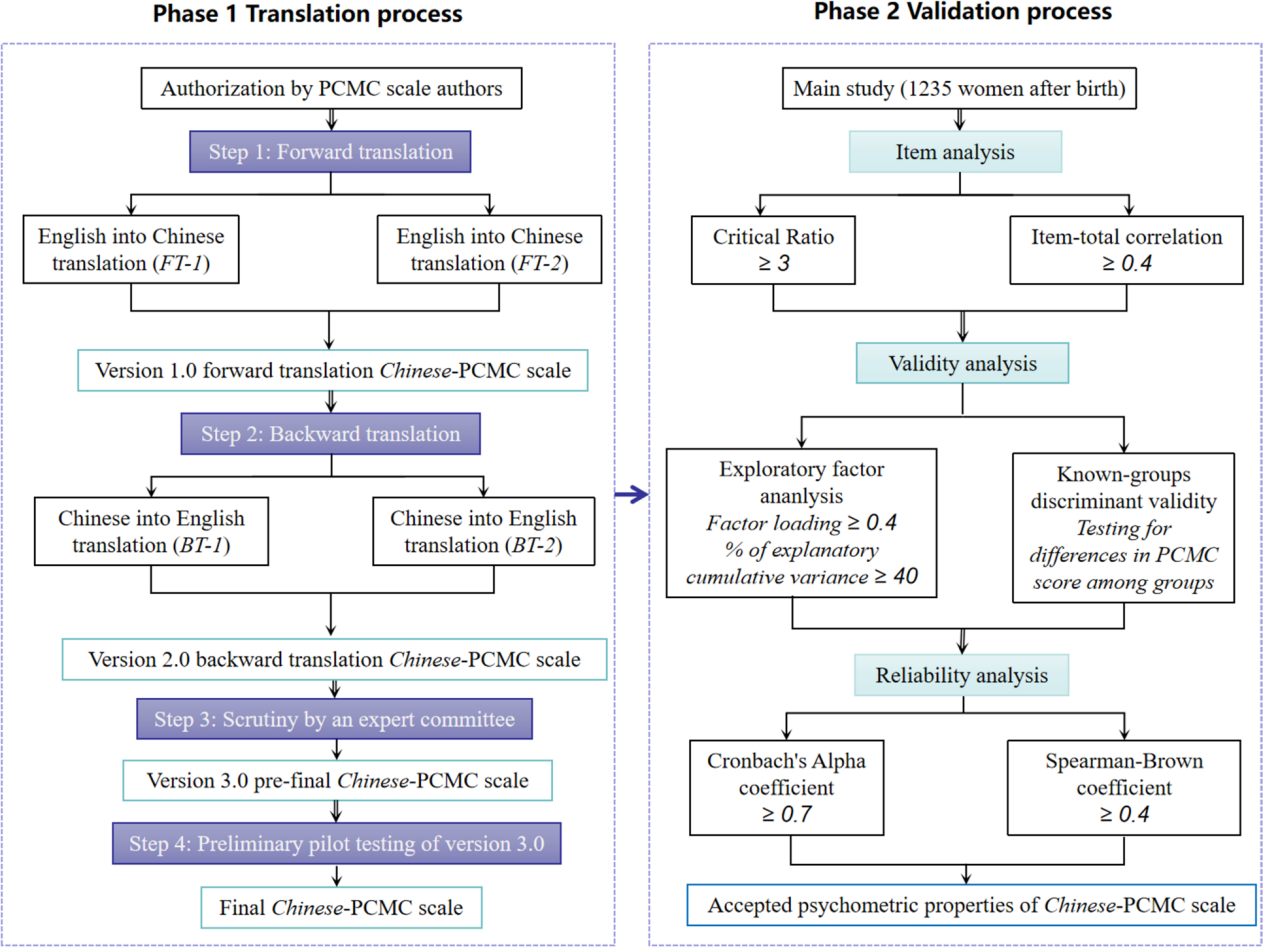
Translation and validation process of the PCMC scale Note: FT-1=Forward translation version-1; FT-2=Forward translation version-2; BT-1=Backward translation version-1; BT-2=Backward translation version-2

This is a descriptive, cross-sectional study. This investigation was divided into two phases (Fig. 1). In phase 1, the PCMC was translated to Chinese using the Beaton intercultural debugging guide [33]. In phase 2, the reliability and validity of the Chinese version of the PCMC were assessed through a cross-sectional survey.

### Translation process

Considering some items in the original scale are not relevant to the Chinese context (such as item 29: “Was there water in the facility?” And item 30 “Was there electricity in the facility?” At present, there is water and electricity in all Chinese facilities),the present study used the 35-item version of the PCMC that was validated in the United States. The US-PCMC is divided into three domains: communication and autonomy, responsive and supportive care, and dignity and respect [26]. The full PCMC score is standardized to range from 0 to 100, with higher scores indicating a more positive birth experience. Permission to translate and validate the PCMC was obtained from the original developers of the scale. The cross-cultural adaptation process followed the Beaton intercultural debugging guidelines, which comprised of forward and backward translations, scrutiny by an expert committee, and preliminary pilot testing [33] ( see Table 1).

- *Step 1: Forward translation:* The 35-item US-PCMC was independently translated into Chinese by two bilingual experts (a midwife and a doctor of evidence-based medicine), both of whom were proficient in both English and native Chinese. A panel of one nursing professor, two nursing postgraduates, and one obstetrician reviewed the forward-translated versions to determine the most accurate translation. Following the resolution of ambiguities and disagreements, a preliminary initial translation version titled “Version 1.0 forward translation Chinese-PCMC” was created.
- *Step 2: Backward translation:* This team consisted of one English teacher and one doctor of nursing, neither of whom had been exposed to the original PCMC. The two researchers translated Version 1.0 into English and named it “Version 2.0 backward translation Chinese-PCMC,” which was then compared to the original PCMC.
- *Step 3: Scrutiny by an expert committee:* Ten experts were invited to evaluate the cultural adaptation of the Version 2.0 *Chinese*-PCMC, which served as the foundation for the Version 3.0 pre-final *Chines*e-PCMC.
- *Step 4: Preliminary pilot testing:* Convenience sampling was used to select (n=30) postnatal women to participate in a preliminary survey that resulted in the final *Chinese*-PCMC. These pilot participants were asked if they had an unclear understanding of the content, and none declared that they did.

**Table 1:** The *Chinese* -Person-centered Maternity Care Scale

Dimension	Items	English Version	Chinese Version
Communication & Autonomy 沟通&自主	2.Introduction 自我介绍	Did each new provider introduce themselves to you when they first came to see you?	与医护人员的首次见面, 他们做过自我介绍吗?
	6.Felt heard 认真倾听	Did you feel heard and listened to by your providers?	你觉得与医务人员交流时, 他们认真倾听了吗?
	9.Involved in decisions 参与决策	Did your providers involve you in decisions about your care?	医护人员曾让您参与医疗决策吗?
	10.Coercion 威逼欺骗	Did you feel coerced or pressured into a decision by providers?	您觉得医务人员曾威逼或欺骗您, 做出医疗决策吗?
	11.Explain procedures 操作告知 (产妇)	Did your providers explain to you why they were doing examinations or procedures on you?	在对您做检查或操作前, 医护人员跟您解释做这个项目的原因吗?
	12.Explain baby procedures 操作告知 (宝宝	Did your providers explain to you why they were doing examinations or procedures on your baby?	在对您的宝宝做操作/检查前, 医护人员跟您解释做这个项目的原因吗?
	13.Consent 知情同意	Did providers or other staff ask your permission/consent before touching or doing procedures or examinations on you?	在做操作/检查前, 医护人员征求过您的许可/同意吗?
	14.Birth preferences respected 生育计划或偏好	Did you feel your birth plan or preferences were respected? (e.g., moving during labor, pain management, music, birthing position)	您觉得医护人员尊重您的生育计划或偏好吗? (例如, 在分娩期间的移动、疼痛管理、音乐、分娩姿势)
	15.Birth position of choice 自由分娩	Were you able to give birth in the position of your choice?	您能自己选择体位分娩吗? (自由体位: 根据自我需求选择生产体位, 如跪趴、直立、趴在分娩球上、面向椅背的坐位、侧躺等)
	16.Language understood 语言	Did your providers speak to you using language or words you could understand?	医护人员用简洁易懂的语言与您交流吗?
	17.Felt informed 分娩经历感知	Did you feel informed about what was happening to you during your childbirth?	您知道生产期间发生的事情吗?
	19.Checked understanding 耐心解释	Did providers check that you understood information that was given to you?	当您向医务人员提问时, 他们耐心解释清楚了吗?
	32.Baby feeding choice respected 喂养方式	Was your feeding choice for your baby (e.g., breastfeeding, bottle feeding, both) respected by providers?	您觉得医护人员尊重您的喂养方式吗? (例如, 母乳喂养、混合喂养和人工喂养)
Supportive Care 支持性照护	1.Wait time 等待时间	How did you feel about the amount of time you had to wait before being examined by a health care provider (doctor or midwife)?	对于产检前等待的时间, 您觉得?
	18.Emotional well-being 心理状态	Did your providers ask about your emotional well-being?	医护人员关心过您的心理状态吗?
	21.Companionship 陪护许可	Were you allowed to have everyone you wanted (e.g., doula, elder, friends, or family) stay with you during your childbirth?	生产期间, 允许长辈、家人、朋友或助产士等陪伴您吗?
	22.Timely response 及时帮助	Did you feel your providers responded in a timely manner when you requested assistance?	当您请求帮助时, 医护人员及时帮助您了吗?
	23.Believed about pain 疼痛信任	Did you feel your providers believed you when you said you were in pain?	当您告诉医务人员您感到疼痛时, 他们相信您吗?
	24.Pain management 疼痛管理	Do you feel your providers did everything they could to help you manage your pain?	医护人员采取措施, 帮助您缓解疼痛过吗?
	29.Took best care 全面照护	Did you feel your providers took the best care of you?	您觉得医护人员照护好您了吗?
	30.Trust 信任	Did you feel you could completely trust your providers with regards to your care?	您信任医护人员对您的照护吗?
	33.Support for baby feeding 喂养支持	Did you receive the support you needed to reach your baby’s feeding goals? (e.g., lactation support)	您得到了实现宝宝喂养目标所需的支持吗? (例如哺乳支持)
	34.Comfortable birth environment 环境舒适	Were you supported in creating a birth environment that made you feel comfortable?	产房及产后病房的环境让您感到舒适吗?
	35.Felt safe 安全感	In general, did you feel physically safe in or around your place of birth?	总体而言, 在产房及产后病房, 您有安全感吗?
Dignity & Respect 尊严&尊重	3.Treated with respect 产妇尊重	Did your providers treat you with respect?	您觉得医护人员尊重您吗?
	4.Experience valued 阅历学识	Did you feel your experience and knowledge were valued?	您觉得医务人员尊重您的阅历和学识吗?
	5.Customs respected 文化习俗	Did you feel your customs and culture were respected by your providers?	您觉得医院人员尊重您的文化习俗吗?
	7.Privacy-covered 隐私保护	During examinations, were you covered up with a cloth or blanket or screened with a curtain so that you did not feel exposed?	做检查时, 为保护隐私, 医护人员用衣物、被套或隔帘为您遮挡过吗?
	8.Information confidential 信息保密	Did you feel your health information was kept confidential and private by providers and staff?	您觉得医务人员会对您的医疗信息保密吗?
	20.Family respected 陪护尊重	Did providers respect your family or companions who were with you?	您觉得医务人员尊重您的家人或陪伴人员吗?
	25.Neglected 忽视	Did you feel your providers avoided, ignored, or otherwise neglected you?	您觉得医务人员忽略、忽视或回避过您吗?
	26.Verbal abuse 语言暴力	Did you feel your providers shouted at you, scolded, insulted, threatened, or talked to you rudely?	医护人员曾对您说话粗鲁过吗?如大喊大叫、辱骂、威胁
	27.Physical abuse 躯体暴力	Did you feel like your providers handled you roughly, held you down, or physically restrained you?	医务人员曾粗暴的对待过您吗?如被推搡、挤压、身体约束
	28.Bribes 贿赂	Did the doctors, nurses or other staff at the facility ask you or your family for money other than the official cost?	医护人员收取过除医疗费用外的钱吗? (红包)
	31.Discrimination 歧视	Would you say you were discriminated against because of your race, ethnicity, culture, sex, gender, sexual orientation, language, immigration status, religion, income, education, age, marital status, number of children, insurance status, or other attribute?	您觉得您被医务人员歧视过吗?因为经济收入、文化水平、语言差异、婚姻状况、生育孩子数量、生育年龄、性取向、民族、宗教信仰、保险类型等

### Sample size

The sample size was calculated according to the criteria required for factorial analysis, with ten to twenty subjects per item [34]. Given the US-PCMC includes 35 items, a sample size of 350 to 700 participants was considered adequate. A total of 1300 women agreed to participate in the study. but 65 participants were excluded because their data was insufficient or unreliable. A sample of 1235 women were therefore included in the data analysis.

## Instruments

### Demographic characteristics form

The following demographic data were collected: age, ethnic group, religion, education level, marital status, parity, mode of delivery, type of maternity wards (double room and single room for Labor, Delivery, Recovery), pregnancy complications, and neonatal complications.

Person-Centered Maternity Care Scale (PCMC).

The 35-item PCMC scaled translated into Chinese was administered in the survey.

### Data collection

Women who gave birth in the preceding six to eight weeks in the postpartum clinics of two tertiary hospitals in Sichuan Province, China, were recruited between December 2022 and January 2023. (Although the recommended time period for postnatal checkups is within the first six weeks after giving birth, most Chinese women visit postpartum clinics at six to eight weeks postpartum). A paper questionnaire was used to collect the data. Participants were informed of the study when they were in the waiting room. Those who agreed to participate signed the informed consent form, and completed the questionnaires by themselves before been seen for postnatal care.

### Data analysis

Data analysis was performed using IBM SPSS Statistics for Windows, Version 21.0 and IBM AMOS Statistics for Windows, Version 24.0. All statistical tests were two-tailed, and a *p*-value of less than 0.05 was considered statistically significant.

### Demographic characteristics

The variables were summarized using frequency and percentages were for the categorical variables, and mean and standard deviations (*SD*) for the continuous variables.

### Content validity

To evaluate the content validity of the PCMC, ten specialists assessed the necessity of each item using a 3-point rating scale. Scale-Content Validity Index (S-CVI) and Item-Content Validity Index (I-CVI) was calculated [35].

### Item analysis

The critical ratio and correlation coefficient methods were used based on item analysis. The item scores on the PCMC were first summed and then arranged in ascending order from high to low. The bottom 27% of the score was classified as the low score group (327 cases) and the top 27% was classified as the high score group (358 cases) [36], and the independent sample for t-test was used to compare the two groups. Pearson correlation coefficients and total scores were then obtained. An absolute critical ratio value of greater than 3 and item-total correlation coefficient greater than 0.4 indicate items have good differentiation [36].

### Exploratory factor analysis

This involved first conducting the Kaiser-Meyer-Olkin (KMO) and Bartlett spherical tests. A KMO test value greater than 0.6 and a statistically significant (*p*<0.001) Bartlett spherical test statistic indicate that the data is suitable for factor analysis [34]. The principal component analysis and maximum variance orthogonal rotation method were used to extract common factors, the cumulative total variance of retained factors should be greater than 40% [34].

### Known-groups discriminant validity

Known-groups discriminant validity was evaluated by testing for differences in the full PCMC score and sub-scale scores in relation to known-groups of demographic characteristics [37]. The independent sample t-test, one-way analysis of variance (ANOVA), and the Kruskal-Wallis H test were performed to compare the full PCMC score and sub-scale scores between different groups.

### Internal consistency

The Cronbach's α coefficient was used to assess the internal consistency of the PCMC. A Cronbach's α coefficient greater than 0.7 was considered acceptable, 0.6-0.699 as tolerable, 0.500-0.599 as tolerable but low, and less than 0.5 as poor [38]. The odd-even split method was used to assess split-half reliability, with this scale’s items divided into two parts, and the Spearman-Brown coefficients of odd-even items calculated.

## Results

### Demographic characteristics of participants

The analytic sample is 1235 postpartum mothers who completed the 35-item PCMC questions. The mean age of the mothers was 31.39 years (*SD*=3.57; range from 22 to 44), and most women were Han Nationality (95.5%), had University education (93.9), were married (99.2% ), primiparas (77.8%), had C-sections (60.6) and delivered in the ordinary ward (73.8%) (see Table 2).

**Table 2:** Demographic characteristics of participants (*n*=1235)

Variables	n (%)or Mean(SD)
**Age**	31.39 (*3.57*)
**Ethnic group**	
Han nationality	1180 (*95.5*)
Ethnic minorities	55 (*4.5*)
**Education level**	
Junior high school and below	18 (*1.5*)
Senior high school	57 (*4.6*)
University and above	1160 (*93.9*)
**Marital status**	
Marriage	1225 (*99.2*)
Unmarried	6 (*0.5*)
Divorced	4 (*0.3*)
**Parity**	
Primipara	961 (*77.8*)
Multipara	274 (*22.2*)
**Type of delivery**	
Vaginal delivery	487 (*39.4*)
Cesarean delivery	748 (*60.6*)
**Maternity wards**	
Double room	912 (*73.8*)
LDR	323 (*26.2*)

*SD* Standard deviation, *LDR* Single room for Labor Delivery, Recovery

### Content validity

The mean age of the specialists was 45.7 years (SD=7.76; range from 39 to 58); The mean working years of the specialists was 23.5 years (SD=9.69; range from 14 to 38); in terms of job title, 80% specialists are deputy chief nurses and 20% specialists are chief nurses. The result of content validity showed that the I-CVI ranged from 0.80 to 1.00 and the S-CVI of 0.950. The result indicated that the experts confirmed the relevance and clarity of the PCMC.

### Item analysis

Apart from three items(i.e., coercion, physical abuse, and bribes) , the critical ratios of all items were greater than 3 (range from 3.311 to 31.212) and significant (*p*<0.01) between the low and high score groups (Table 3). Apart from that of seven items (i.e., customs respected, coercion, birth position of choice, verbal abuse, physical abuse, bribes, and discrimination), the item-total correlation coefficients were greater than 0.4 and significant (*p*<0.01) (Table 3). Although the critical ratios of items were a little below 3 andt the gap is narrow, we decided to retain all the items in the *Chines*e-PCMC in view of the literature review and expert advice.

**Table 3:** Items analysis of the Person-centered Maternity Care Scale

Item’s label	Groups (Mean±SD)		Critical Ratio	Item-total Correlation
	Low Score Group (*n*=360)	High Score Group (*n*=355)		
1.Wait time	2.14±0.68	2.63±0.56	-10.456**	0.321**
2.Introduction	0.77±0.69	1.87±0.96	-17.562**	0.464**
3.Treated with respect	2.14±0.63	2.96±0.21	-23.563**	0.637**
4.Experience valued	2.14±0.62	2.96±0.21	-23.596**	0.638**
5.Customs respected	2.07±0.42	2.21±0.41	-4.392**	0.190**
6.Felt heard	2.19±0.60	2.98±0.14	-24.519**	0.632**
7.Privacy-covered	2.67±0.57	2.98±0.17	-9.950**	0.385**
8.Information confidential	2.46±0.65	2.96±0.24	-13.825**	0.457**
9.Involved in decisions	1.84±0.88	2.62±0.66	-13.406**	0.398**
10.Coercion	2.94±0.31	2.99±0.16	-2.858**	0.105**
11.Explain procedures	1.52±0.98	2.96±0.19	-27.330**	0.619**
12.Explain baby procedures	1.52±0.98	2.96±0.19	-27.330**	0.621**
13.Consent	2.04±1.02	3.00±0.05	-17.924**	0.523**
14.Birth preferences respected	2.06±0.99	3.00±0.05	-17.943**	0.524**
15.Birth position of choice	1.23±0.87	1.57±0.65	-5.975**	0.172**
16.Language understood	2.25±0.67	2.99±0.08	-20.943**	0.597**
17.Felt informed	2.00±0.66	2.99±0.08	-28.257**	0.700**
18.Emotional well-being	1.66±0.78	2.95±0.21	-30.430**	0.709**
19.Checked understanding	1.99±0.68	2.99±0.08	-27.892**	0.703**
20.Family respected	2.20±0.66	2.97±0.17	-21.294**	0.615**
21.Companionship	1.95±1.10	2.82±0.53	-13.558**	0.374**
22.Timely response	2.26±0.59	2.98±0.13	-22.632**	0.627**
23.Believed about pain	2.47±0.62	2.97±0.18	-14.517**	0.464**
24.Pain management	2.11±0.67	2.91±0.32	-20.537**	0.526**
25.Neglected	2.51±0.75	2.98±0.18	-11.716**	0.395**
26.Verbal abuse	2.92±0.37	3.00±0.05	-3.965**	0.182**
27.Physical abuse	2.98±0.18	3.00±0.00	-1.725	0.064*
28.Bribes	2.99±0.16	3.00±0.00	-0.993	0.014
29.Took best care	2.12±0.51	2.98±0.14	-31.115**	0.677**
30.Trust	2.39±0.55	3.00±0.05	-20.565**	0.605**
31.Discrimination	2.96±0.26	3.00±0.00	-3.311**	0.091**
32.Baby feeding choice respected	2.12±0.50	2.98±0.14	-31.212**	0.676**
33.Support for baby feeding	2.40±0.55	3.00±0.05	-20.638**	0.602**
34.Comfortable birth environment	1.76±0.84	2.72±0.60	-17.665**	0.454**
35.Felt safe	2.13±0.72	2.91±0.28	-19.336**	0.544**

*P≤0.05

**P≤0.01

### Exploratory factor analysis

Exploratory factor analysis found the KMO value to be 0.828, and the Bartlett spherical test statistic to be 48157.862 (*p*<0.001), thus demonstrating that the data was suitable for factor analysis. We decided to limit the number of extracted common factors was to 3, explaining a total variance of 40.803% (communication & autonomy, 23.353%; supportive care, 11.171%; dignity & respect, 6.279%). Apart from five items (i.e., coercion, birth position of choice, wait time, customs respected, discrimination), the loading value on the corresponding common factor for the remainder of the items was greater than 0.3 (Table 4).

**Table 4:** Exploratory factor analysis of the Person-centered Maternity Care Scale

Scale Items	Factor Loading			Common Factor Variance
	Factor 1	Factor 2	Factor 3	
2.Introduction	0.416			0.183
6.Felt heard	0.660			0.451
9.Involved in decisions	0.355			0.140
10.Coercion	0.095			0.013
11.Explain procedures	0.534			0.830
12.Explain baby procedures	0.536			0.832
13.Consent	0.442			0.816
14.Birth preferences respected	0.443			0.809
15.Birth position of choice	0.099			0.046
16.Language understood	0.609			0.396
17.Felt informed	0.744			0.571
19.Checked understanding	0.747			0.574
32.Baby feeding choice respected	0.734			0.602
1.Wait time		0.286		0.083
18.Emotional well-being		0.720		0.530
21.Companionship		0.334		0.133
22.Timely response		0.666		0.476
23.Believed about pain		0.487		0.264
24.Pain management		0.535		0.309
29.Took best care		0.736		0.603
30.Trust		0.664		0.549
33.Support for baby feeding		0.661		0.546
34.Comfortable birth environment		0.443		0.237
35.Felt safe		0.561		0.383
3.Treated with respect			0.670	0.472
4.Experience valued			0.672	0.473
5.Customs respected			0.175	0.033
7.Privacy-covered			0.395	0.157
8.Information confidential			0.476	0.240
20.Family respected			0.638	0.429
25.Neglected			0.415	0.207
26.Verbal abuse			0.524	0.329
27.Physical abuse			0.861	0.758
28.Bribes			0.883	0.796
31.Discrimination			0.080	0.013
Eigenvalue (before rotated)	9.288	2.866	2.127	—
% of explanatory variance (before rotated)	26.537	8.189	6.077	
% of explanatory cumulative variance (before rotated)	26.537	34.726	40.803	
Eigenvalue (rotated)	8.174	3.910	2.197	
% of explanatory variance (rotated)	23.353	11.171	6.279	
% of explanatory cumulative variance (rotated)	23.353	34.524	40.803	

### Known-groups discriminant validity

The PCMC total score was related to type of delivery and maternity wards, with higher scores among those who delivered by c-sections and those who delivered in a single room for Labor, Delivery, Recovery compared to those who delivered vaginally and in the general ward respectively (Table 5).

**Table 5:** Differences in the Person-Centered Maternity Care Scale score between known-groups (*n*=1235)

Variables	Number	Standardized scores (Mean±SD)	Statistics	*P*-Value
**Ethnic group**				
Han nationality	1180	84.77±9.75	-1.674^a^	0.094
Ethnic minorities	55	87.03±10.30		
**Education level**				
Junior high school and below	18	86.13±9.39	1.058^b^	0.367
Senior high school	57	87.40±8.73		
University and above	1160	85.24±9.86		
**Marital status**				
Marriage	1225	84.84±9.79	0.829^b^	0.799
Unmarried	6	88.73±8.02		
Divorced	4	89.52±7.97		
**Parity**				
Primipara	961	84.68±9.90	-1.286^a^	0.199
Multipara	274	85.54±9.35		
**Type of delivery**				
Vaginal delivery	487	83.78±9.65	-3.17^a^	0.002**
Cesarean delivery	748	85.58±9.81		
**Maternity wards**				
Double room	912	84.44±10.03	-2.764^a^	0.006**
LDR	323	86.09±8.94		

SD Standard deviation

*P<0.05

**P<0.01

^a^Independent sample t-test, t

^b^one-way analysis of variance (ANOVA), F; LDR, Single room for Labor, Delivery, Recovery

### Internal consistency

The Cronbach’s alpha coefficient of the full set of PCMC was 0.989, with that of the subscale ranging from 0.669 to 0.840. The Spearman-Brown coefficient of the full PCMC was 0.852, with that of the subscales ranging from 0.449 to 0.798 (Table 6).

**Table 6:** Cronbach's Alpha coefficient and Spearman-Brown coefficient of Person-centered Maternity Care Scale (*n*=1235)

Variables	Number of Items	Summative scores (Mean±SD)	Standardized score (Mean±SD)	Cronbach's Alpha coefficient	Spearman-Brown coefficient
Communication & autonomy	13	30.85±5.35	79.11±13.73	0.822	0.798
Supportive care domain	11	27.92±4.22	84.61±12.77	0.840	0.734
Dignity & respect	11	30.34±2.33	91.95±7.06	0.669	0.449
Total of PCMC	35	89.12±10.27	84.87±9.78	0.898	0.852

*SD* Standard Deviation

## Discussion

A growing body of evidence reveals that the mistreatment of pregnant women during facility-based childbirth occurs across the globe [39]. The aim of the current study was to evaluate the psychometric properties of PCMC in Chinese postpartum women. The findings showed that the Chinese version of the PCMC had robust validity and reliability for assessing the level of maternity care in the multicultural context of China.

The findings of the item analysis showed that these items, which include customs respected, coercion, birth position of choice, verbal abuse, physical abuse, bribes, and discrimination, exhibited poor discrimination between the low score and high score groups. In terms of exploratory factor analysis, 3 factors explained a total variance of 40.803% that was higher than the recommended value (40%), but the questions on customs respected, coercion, birth position of choice, and discrimination had loading values lower than 0.3. These findings were not consistent with the US validation findings [26], which is likely due to discrepancies in the sample distribution. On the one hand, the site of delivery is affiliated with the *Chinese National Health and Family Planning Commission*; hence, the quality of health services is higher compared to other primary hospitals. On the other hand, 95% of the participants in the present study were Han nationals with no specific cultural customs. Thus, the items with poor psychometric properties (customs respected, coercion, birth position of choice, verbal abuse, physical abuse, bribes, discrimination) were still retained in view of the literature review and expert advice.

Regarding known-groups discriminant validity, we found that on average, women who had Caesarean sections had a higher PCMC score compared to women who had vaginal deliveries. In the study, 60.6% women has a cesarean delivery and 39.4% had a vaginal delivery. This high c-section rate may be explained by the site of delivery being affiliated with the Regional medical center*,* with most participants being from southwest China, and having high-risk pregnancy factors, which results in high cesarean section numbers. The higher PCMC scores may be due to a greater attention to the experiences of such patients. It is noteworthy that a statistically significant difference was found in PCMC scores by the type of maternity ward, with women delivering in a single room for labor, delivery, and recovery having higher PCMC than those delivering in double room. A potential explanation may be that the single room promotes birth as a normal family process, leading to a greater level of Person-Centered Maternity Care through privacy provisions and other aspects of Person-Centered Maternity Care [40].

Concerning internal consistency, apart from the Cronbach’s alpha coefficient of the dignity and respect sub-scale that was tolerable, the Cronbach’s alpha coefficient of the full PCMC and other sub-scales exceeded the value of 0.7, which is acceptable. The split-half reliability of the PCMC was also acceptable, indicating stability over time. In general, the result of the current study found that the Chinese version of PCMC had good internal consistency, which is consistent with previous studies [8, 24, 27].

Although this study used a strong scientific approach with robust methods to translate and investigate the performance of the PCMC in a Chinese context, there are some limitations. Firstly, the women recruited for this study came from only two tertiary hospitals in Sichuan Province, and were quite homogeneous—mostly of Han nationality and with high education and married. Also, about 61% delivered by c-section. Thus, this sample is not representative of other populations in China. However, the construct of the PCMC is likely generic, irrespective of geographical location and patient characteristics.

## Conclusions

This study demonstrated the robust psychometric properties of the PCMC, revealing that it is a reliable and valid tool for evaluating Person-Centered Maternity Care in a Chinese context. The Person-Centered Maternity Care Scale can now be used by those working with Chinese-speaking populations as an objective and robust measure. Moreover, it will be a valuable tool for understanding aspects of Person-Centered Maternity Care that need to be addressed in interventions as well as aid in the evaluation of interventions.

## Data Availability

The datasets used and/or analyzed during the current study available from the corresponding author on reasonable request.

## Abbreviations

WHO: World Health Organization
PTSD: Post-traumatic Stress Disorder
CEQ-2.0: Childbirth Experience Questionnaire 2.0 version
WDEQ-A and WDEQ-B: Wijma Delivery Expectancy Questionnaire version A and B
PCMC Scale: Person-Centered Maternity Care (PCMC) Scale
SD: Standard Deviations
KMO: Kaiser-Meyer-Olkin
S-CVI: Scale-Content Validity Index
I-CVI: Item-Content Validity Index
ANOVA: One-Way Analysis of Variance
